# Celastrol protects against early brain injury after subarachnoid hemorrhage in rats through alleviating blood-brain barrier disruption and blocking necroptosis

**DOI:** 10.18632/aging.203221

**Published:** 2021-06-28

**Authors:** Hangzhe Xu, Yong Cai, Mengyan Yu, Jing Sun, Jing Cai, Jingbo Li, Bing Qin, Guangyu Ying, Ting Chen, Yongfeng Shen, Liyong Jie, Demin Xu, Chi Gu, Chun Wang, XiaoYi Hu, Jingsen Chen, Lin Wang, Gao Chen

**Affiliations:** 1 Department of Neurosurgery, The Second Affiliated Hospital of Zhejiang University, School of Medicine, Hangzhou 310016, China; 2 School of Medicine, Zhejiang University, Hangzhou 310012, China; 3 Neurointensive Care Unit, The Second Affiliated Hospital of Zhejiang University, School of Medicine, Hangzhou 310016, China; 4 Department of Neurosurgery, Hangzhou First People’s Hospital, Hangzhou 310006, China; 5 Department of Radiology, The Second Affiliated Hospital of Zhejiang University, School of Medicine, Hangzhou 310016, China; 6 Department of Radiology, Peking University Shenzhen Hospital, Shenzhen 518034, China

**Keywords:** subarachnoid hemorrhage, early brain injury, celastrol, blood-brain barrier, necroptosis

## Abstract

Background: Subarachnoid hemorrhage (SAH) is a life-threatening disease worldwide, and effective pharmaceutical treatment is still lacking. Celastrol is a plant-derived triterpene which showed neuroprotective potential in several types of brain insults. This study aimed to investigate the effects of celastrol on early brain injury (EBI) after SAH.

Methods: A total of sixty-one male Sprague-Dawley rats were used in this study. Rat SAH endovascular perforation model was established to mimic the pathological changes of EBI after SAH. Multiple methods such as 3.0T MRI scanning, immunohistochemistry, western blotting and propidium iodide (PI) labeling were used to explore the therapeutic effects of celastrol on SAH.

Results: Celastrol treatment attenuated SAH-caused brain swelling, reduced T2 lesion volume and ventricular volume in MRI scanning, and improved overall neurological score. Albumin leakage and the degradation of tight junction proteins were also ameliorated after celastrol administration. Celastrol protected blood-brain bairrer integrity through inhibiting MMP-9 expression and anti-neuroinflammatory effects. Additionally, necroptosis-related proteins RIP3 and MLKL were down-regulated and PI-positive cells in the basal cortex were less in the celastrol-treated SAH group than that in untreated SAH group.

Conclusions: Celastrol exhibits neuroprotective effects on EBI after SAH and deserves to be further investigated as an add-on pharmaceutical therapy.

## INTRODUCTION

Subarachnoid hemorrhage (SAH) is a life-threatening neurologic emergency with a mortality rate reaching nearly 50% around the world and accompanied by heavy burden on patients’ families and health care systems [1, 2]. Early brain injury (EBI) is considered the main cause of poor prognosis of SAH patients and is becoming a hot area of research [3, 4]. Although pharmacotherapies were actively developed and aimed to attenuate EBI after SAH, effective pharmaceutical drugs for SAH patients are still waiting to be found.

In recent years, botanical compounds have drawn increasing attention in the management of SAH, such as curcumin [5] and resveratrol [6]. Celastrol (Cel) is a plant-derived triterpenoid which was isolated from the root bark of the traditional Chinese herb “Thunder of God Vine” (Tripterygium wilfordii Hook F.) [7]. Previous studies have demonstrated the promising effects of celastrol on auto-immune diseases and chronic inflammation [7–9]. Furthermore, researchers also discovered the anti-tumor activity of celastrol both *in vitro* and *in vivo* [10–12]. Celastrol’s neuroprotective effects were also observed in neurodegenerative diseases [13, 14], traumatic brain injury [15] and ischemic brain injury [16]. However, celastrol’s neuroprotective effects on EBI after SAH has not been reported yet. Luo et al. [17] demonstrated that celastrol could recover the transepithelial electrical resistance loss and protected tight junction proteins (ZO-1, occluding and claudin-5) from degradation in an *in vitro* oxygen glucose deprivation (OGD) model of endothelial bEnd3 cells. Blood-brain barrier (BBB) disruption was regarded as the main cause of brain edema in EBI and was associated with poor prognosis after SAH [18]. This study also aimed to investigate to ability of celastrol on suppressing BBB disruption *in vivo* and its neuroprotective role after SAH.

Necroptosis, also known as programmed necrosis, was firstly discovered and named by Degterev et al. in 2005, as a new form of cell death in ischemic brain injury [19]. After that, this kind of programmed cell death was also discovered in TBI [20], spinal cord injury (SCI) [21] and intracerebral hemorrhage [22]. Recent studies have discovered the existence of necroptosis after SAH [23]. Researchers also found that celastrol could inhibit necroptosis through suppressing the RIP3/MLKL signaling pathway in a mouse ulcerative colitis model [24]. Herein, we hypothesized that necroptosis could be a potential therapeutic target in EBI after SAH, and celastrol might exert neuroprotective effects by attenuating the RIP3/MLKL signaling pathway.

In this study, we aimed to use a rat endovascular puncture model as well as MRI scanning to determine whether celastrol could suppress necroptosis and alleviate SAH-related symptoms after SAH.

## RESULTS

### SAH grade, mortality and neurological scores

**Figure 1:**
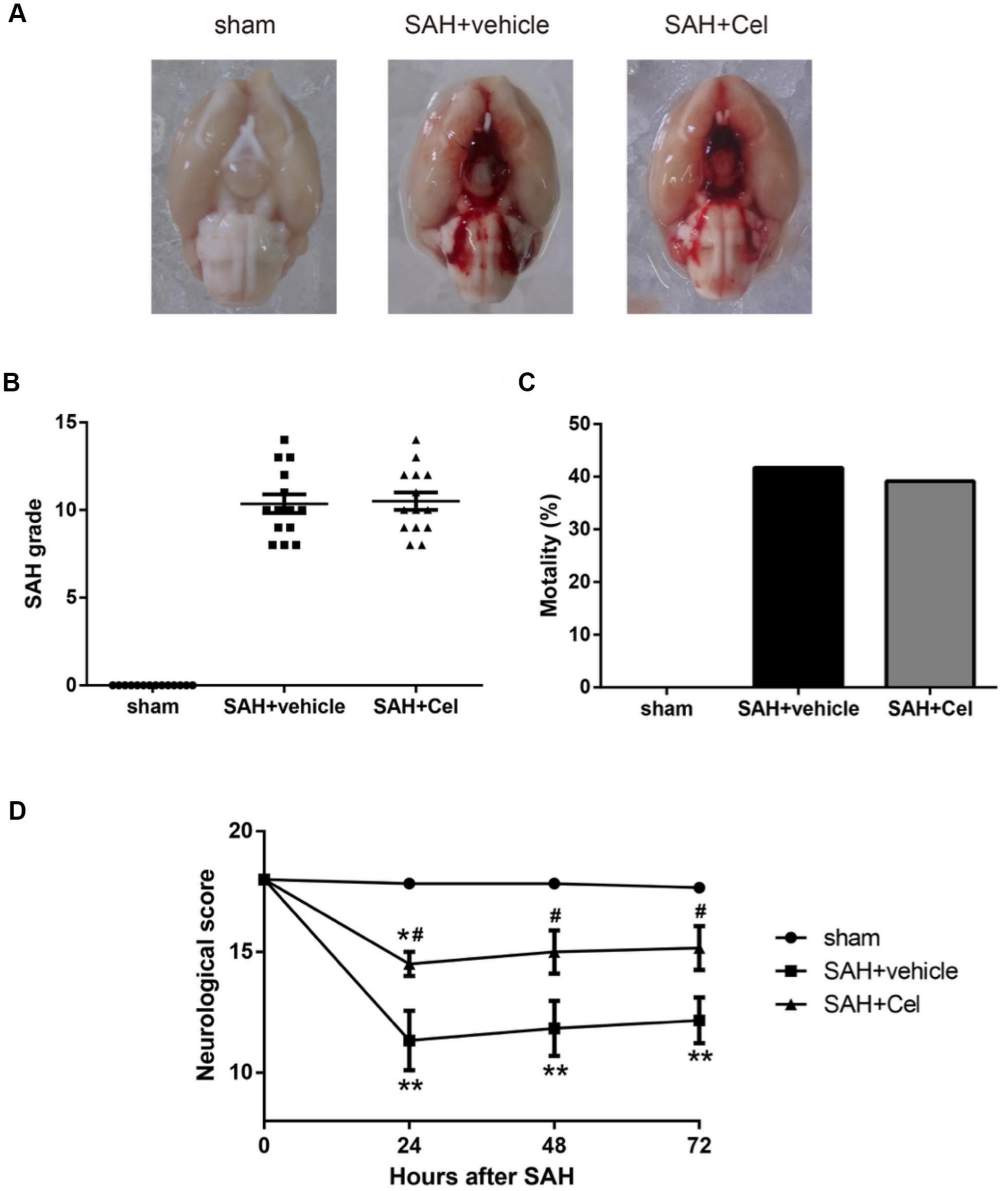
**Representative pictures of brains, SAH grade, mortalities and neurological scores at 72 h after SAH.** (**A**) Typical brains of sham, SAH + vehicle, and SAH + Cel group. (**B**) The grade of SAH severity. (**C**) SAH-caused mortality rate. (**D**) Neurological scores at 24, 48 and 72 h after SAH induction. Data were presented as mean±SEM. *n* = 14. *P < 0.05 versus sham, **P < 0.01 versus sham, ^#^*P* < 0.05 versus SAH + vehicle.

**Figure 2:**
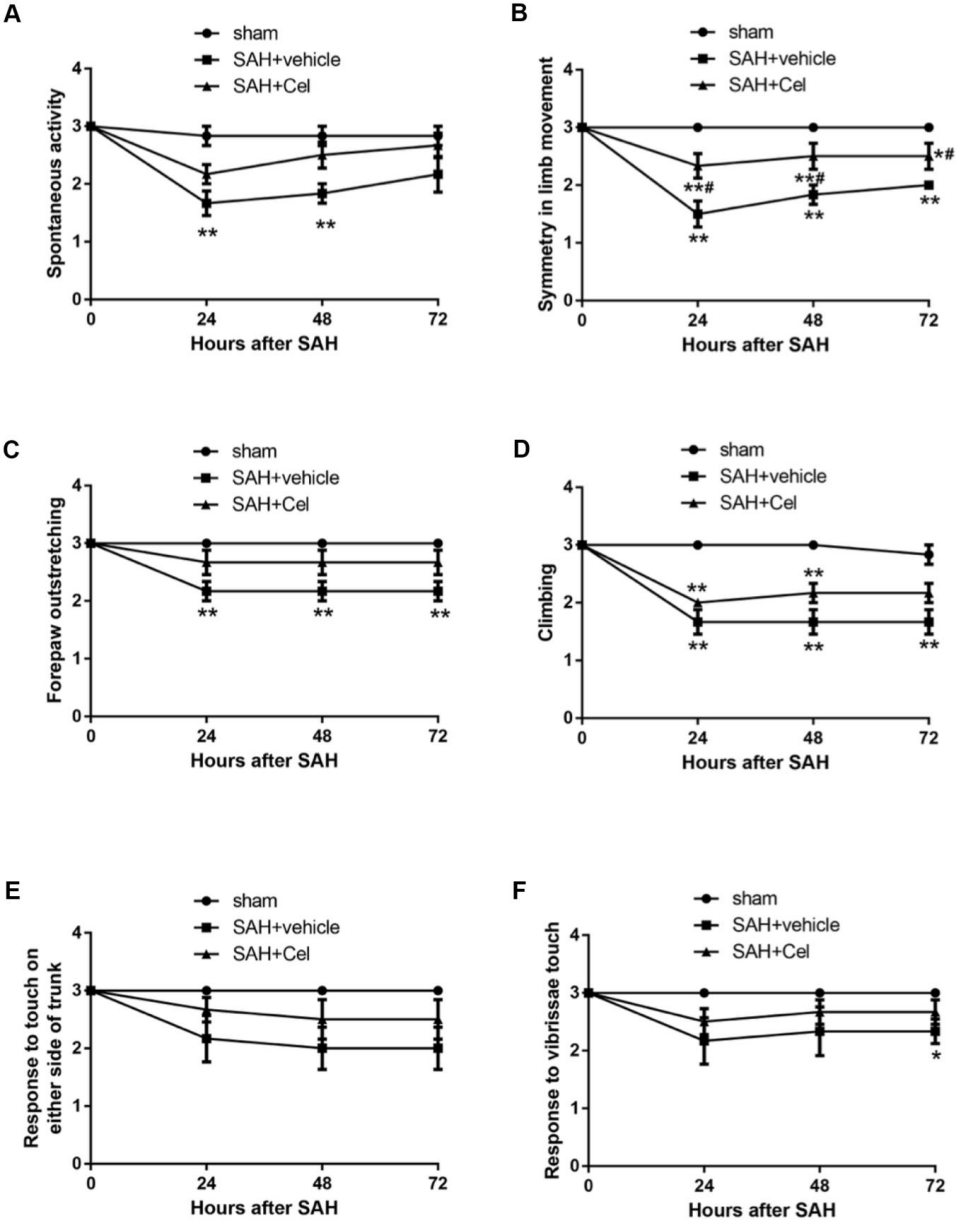
**Neurological subscores at 24, 48 and 72h after SAH induction.** (**A**–**F**) Subscores of (**A**) spontaneous activity, (**B**) symmetry in limb movement, (**C**) forepaw outstretching, (**D**) climbing, (**E**) response to touch on either side of trunk, (**F**) response to vibrissae at each time point after SAH. Data were presented as mean±SEM. n = 14. *P < 0.05 versus sham, **P < 0.01 versus sham, #P < 0.05 versus SAH + vehicle.

Representative brain images were shown in Figure 1A. No significant differences of the SAH grade scores were observed after the treatment of Cel, compared to the vehicle group (*P*>0.05, Figure 1B). The mortality rates were as follows: sham group 0% (0/14), SAH + vehicle group 41.7% (10/24) and SAH + Cel group 39.1% (9/23), which were comparable between SAH + vehicle group and SAH + Cel group (*P*>0.05, Figure 1C). The total neurological score of the SAH + vehicle group was dramatically lower than those in the sham group at 24, 48 and 72h after SAH induction (*P*<0.01, Figure 1D). Treatment with celastrol markedly ameliorated the total neurological deficits at each time point after SAH (*P*<0.05 vs SAH + vehicle, Figure 1D). As for the detailed neurological function (neurological subscores), celastrol significantly improved the symmetry in limb movement at each time point (*P*<0.05 vs SAH + vehicle, Figure 2B).

### Celastrol attenuated brain swelling, reduced T2 lesion volume and ventricular volume after SAH

**Figure 3:**
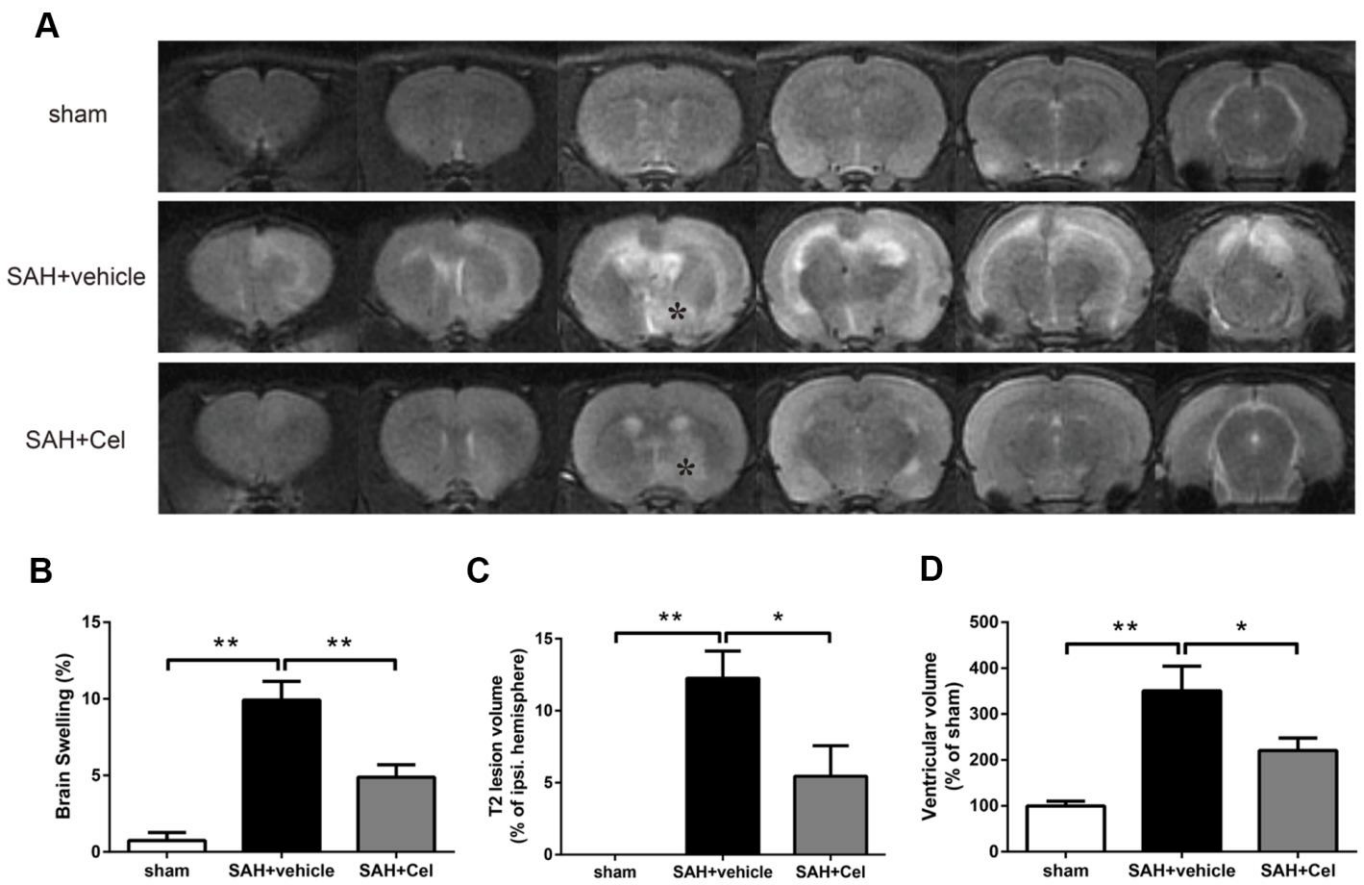
**Celastrol attenuated brain swelling, reduced T2 lesion volume and ventricular volume after SAH.** (**A**) Representative T2-weighted MRI images (3.0T) of the brains of sham, SAH + vehicle, and SAH + Cel group. (**B**) Brain swelling was calculated as: ((volume of ipsilateral hemisphere - volume of contralateral hemisphere)/volume of contralateral hemisphere) × 100%. (**C**) T2 lesion volume was presented as the volume ratio to the ipsilateral hemisphere. (**D**) Ventricular volume was calculated as Σ(A_n_ + A_n + 1_) × d / 2, and was presented as the volume ratio to the average volume of the sham group. Data were presented as mean±SEM. *n* = 6. *P < 0.05, **P < 0.01.

The representative MRI T2 images were shown in Figure 3A. Experimental SAH resulted in a significant brain swelling of the left hemisphere at 72 h (*P*<0.01 vs sham, Figure 3B). Notably, Celastrol pretreatment decreased the symptom of brain swelling of the left hemisphere at 24 h (*P*<0.01 vs SAH + vehicle, Figure 3B). No T2 lesion was observed in ipsilateral hemisphere in the sham group, but it was obvious in SAH + vehicle group (*P*<0.01 vs sham, Figure 3A). In addition, celastrol treatment significantly decreased the T2 lesion volume after SAH (*P*<0.05 vs SAH + vehicle, Figure 3C). The ventricular volumes of the rats were significantly enlarged because of SAH (*P*<0.01 vs sham, Figure 3D), while celastrol had the ability to prevent this process (*P*<0.05 vs SAH + vehicle, Figure 3D).

### Celastrol attenuated albumin leakage after SAH

**Figure 4:**
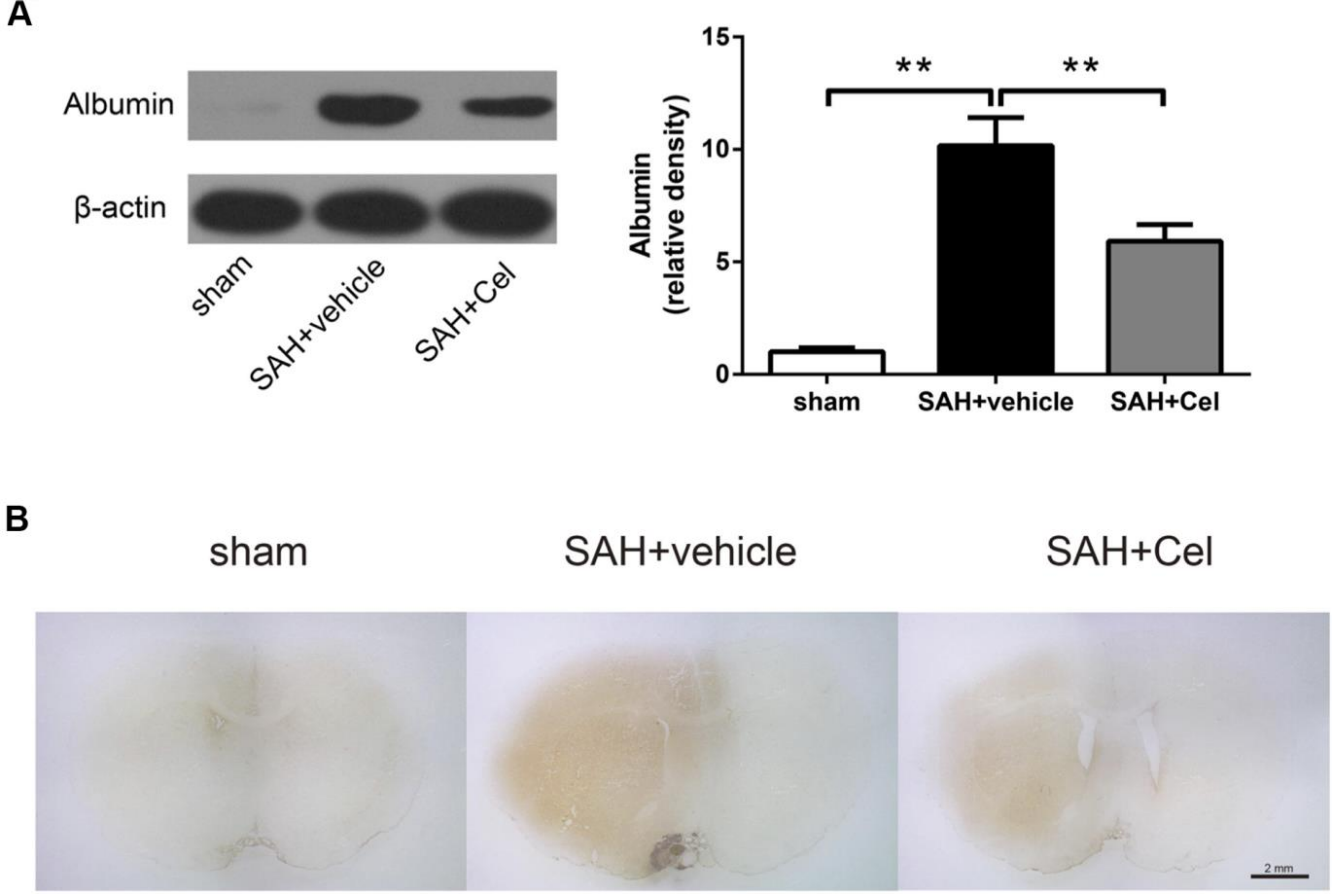
**Celastrol decreased albumin leakage after SAH.** (**A**) Protein levels of albumin in the ipsilateral basal cortex in sham, SAH + vehicle, and SAH + Cel groups at 72 h after SAH induction, detected by WB. (**B**) Representative histological slides of the albumin staining in the perivascular regions of the ipsilateral basal cortex in sham, SAH + vehicle, and SAH + Cel group. Data were presented as mean±SEM. *n* = 6. **P < 0.01. Scale bar = 2 mm.

Western blotting (WB) and immunohistochemistry (IHC) and of albumin were performed to evaluate the disruption of BBB. SAH significantly facilitated the protein leakage of albumin at 72 h (*P*<0.01 vs. sham, Figure 4A), while celastrol markedly prevented this process (*P*<0.01 vs. SAH + vehicle, Figure 4A). Representative photographs of albumin immunoreactivity were presented in Figure 4B.

### Celastrol prevented tight junction protein disruption after SAH

**Figure 5:**
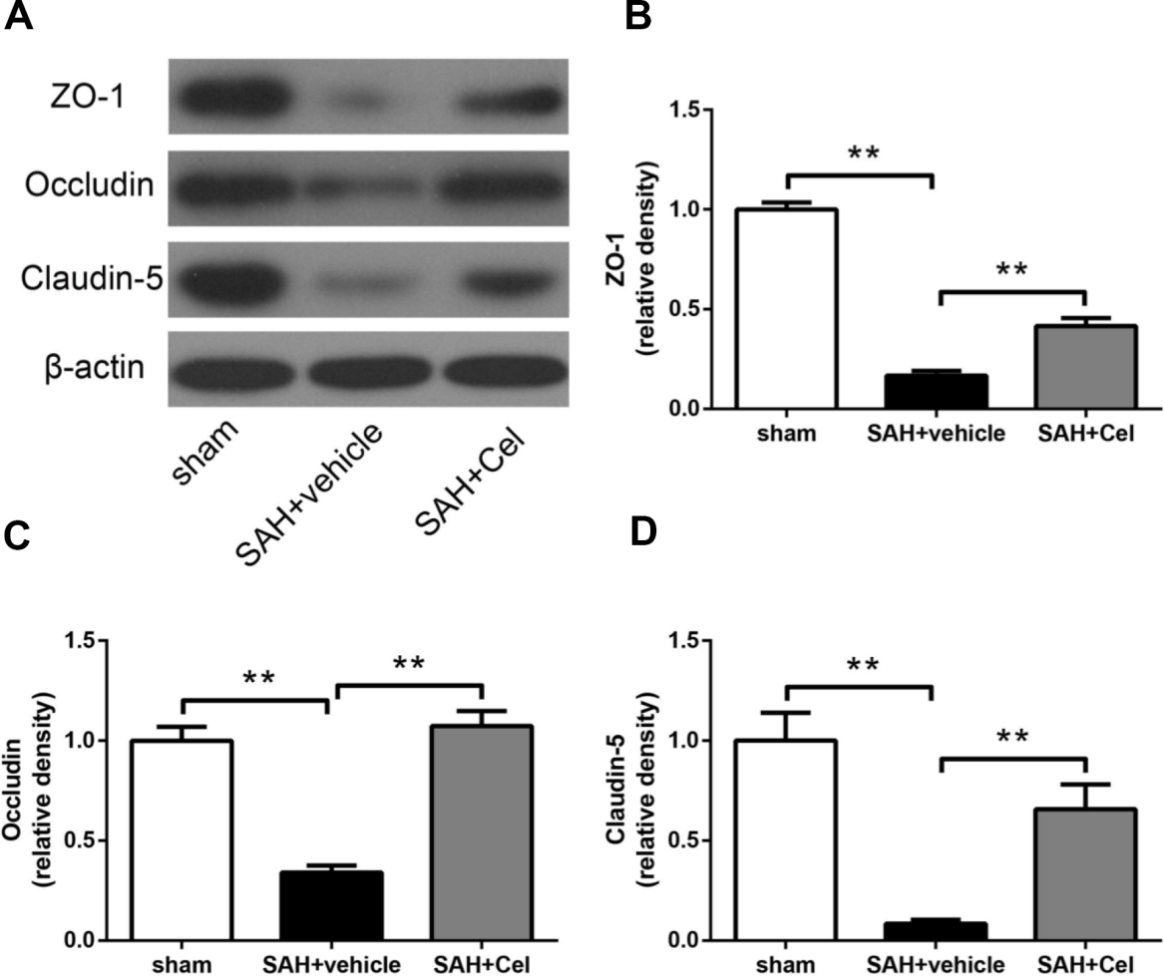
**Effects of celastrol treatment on tight junction proteins at 72 h after SAH induction.** (**A**) Representative western blots showing levels of ZO-1, occludin and claudin-5 in the ipsilateral cortex at 72 h after SAH induction. (**B**–**D**) Quantification of band densities of ZO-1, occludin and claudin-5. The densities of the protein bands were analyzed and normalized to β-actin, and compared to the mean value of the sham group. Data were presented as mean±SEM. *n* = 6. **P < 0.01.

To evaluate the effects of celastrol treatment on tight junction proteins after SAH (72h), WB was performed to investigate the protein expression of occludin, ZO-1 and claudin-5. Significant decrease of these proteins was detected in SAH + vehicle group (*P*<0.01 vs sham, Figure 5A–5D). And celastrol administration remarkably attenuated the decrease of occludin, ZO-1 and claudin-5 (*P*<0.01 vs SAH + vehicle, Figure 5A–5D). These results indicated that celastrol attenuated BBB disruption at 72h after SAH.

### Celastrol prevented the up-regulation of MMP-9 after SAH

**Figure 6:**
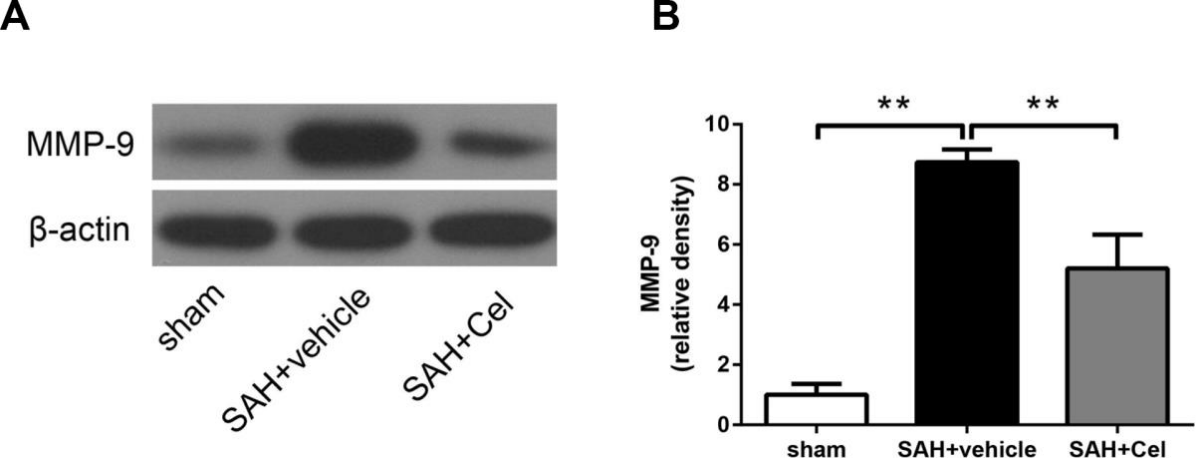
**Effect of celastrol treatment on MMP-9 expression at 72 h after SAH induction.** (**A**) Representative WB showing levels of MMP-9 in the ipsilateral cortex of each group at 72 h after SAH induction. (**B**) Quantifications of band densities of MMP-9. The densities of the protein bands were analyzed and normalized to β-actin, and compared to the mean value of the sham group. Data were presented as mean±SEM. *n* = 6. **P < 0.01.

BBB disruption resulted from tight junction protein degradation after stroke was mediated by matrix metalloproteases (MMPs) [25], especially MMP-9 [26]. MMP-9 expression was significantly up-regulated after SAH induction (*P*<0.01 vs. sham, Figure 6A, 6B), and treatment of celastrol attenuated this process (*P*<0.01 vs SAH + vehicle, Figure 6A, 6B).

### Celastrol attenuated neuroinflammation after SAH

**Figure 7:**
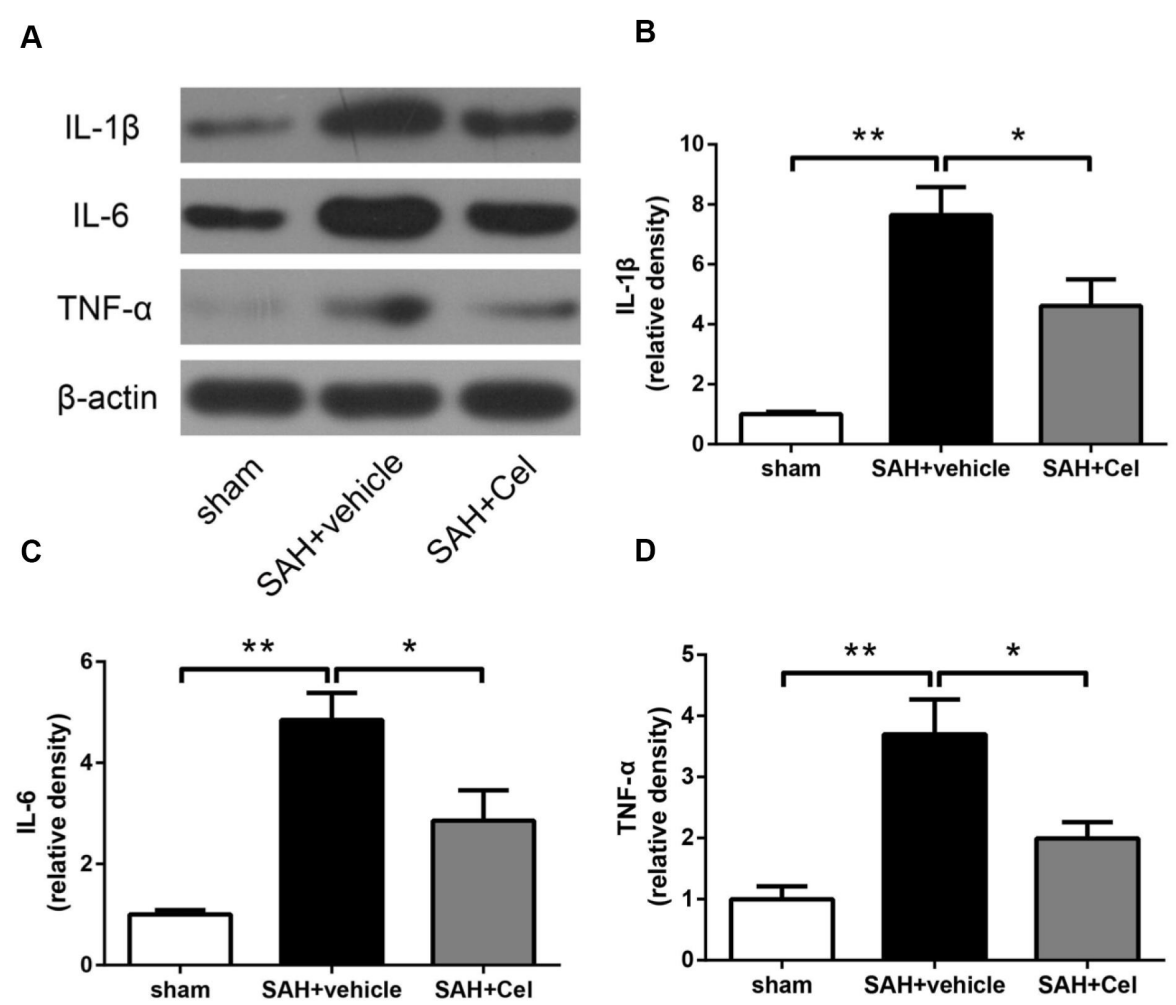
**Celastrol decreased neuroinflammation after SAH induction.** (**A**) Representative WB of protein levels of IL-1β, IL-6 and TNF-α in the ipsilateral cortex in each group at 72 h after SAH induction. (**B**–**D**) The relative band densities of IL-1β, IL-6 and TNF-α. The densities of the protein bands were analyzed and normalized to β-actin, and compared to the mean value of the sham group. Data were presented as mean±SEM. *n* = 6. *P < 0.05, **P < 0.01.

The neuroinflammatory response was considered to be one of the main causes of the up-regulation of MMP-9 after SAH, as reported in previous studies [27, 28]. To further explore the mechanisms of the suppression of MMP-9 after celastrol treatment, we detected the expression of pro-inflammatory cytokines. As presented in Figure 7A–7D, the protein levels of IL-1β, TNF-α and IL-6 were dramatically up-regulated at 72 h after SAH induction (*P*<0.01 vs. SAH + vehicle), but the administration of celastrol prevented the increasing of these cytokines induced by SAH (*P*<0.05 vs SAH + vehicle).

### Celastrol down-regulated RIP3/MLKL-mediated necroptosis after SAH

**Figure 8:**
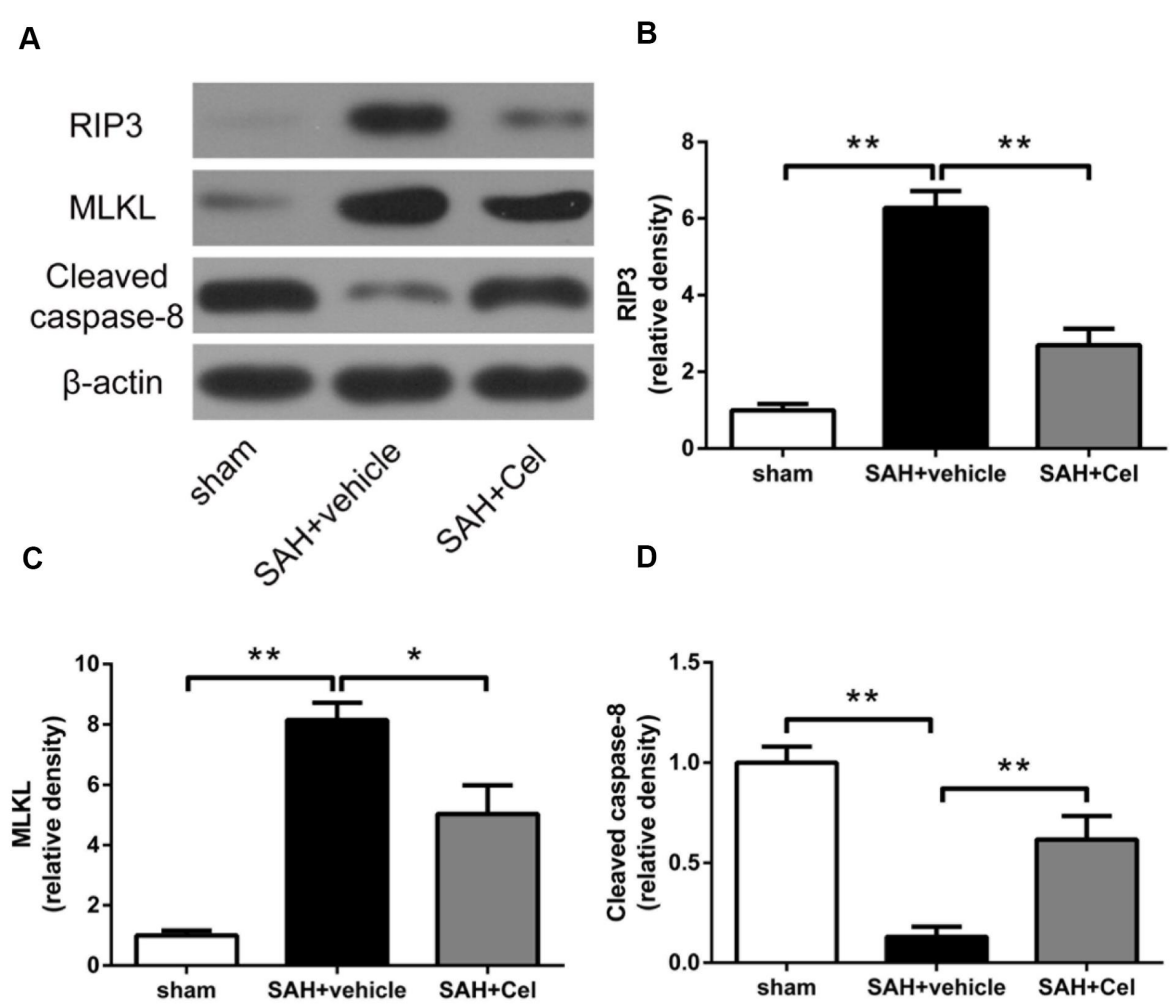
**Celastrol down-regulated RIP3/MLKL signaling pathway after SAH induction.** (**A**) Representative WB showing protein levels of RIP3, MLKL and cleaved caspase-8 in the ipsilateral cortex in each group at 72 h after SAH induction. (**B**–**D**) Protein quantification of RIP3, MLKL and cleaved caspase-8. The densities of the protein bands were analyzed and normalized to β-actin, and compared to the mean value of the sham group. Data were presented as mean±SEM. *n* = 6. *P < 0.05, **P < 0.01.

To further investigate the role of celastrol in necroptosis, WB was conducted to detect the protein expression of the RIP3/MLKL signaling pathway. We found that RIP3 and MLKL expression were significantly upregulated in SAH + vehicle group than that in sham group (*P*<0.01, Figure 8B, 8C), while celastrol administration exerted a prominent inhibitory effects on the expression of these proteins (*P*<0.05 vs SAH + vehicle, Figure 8B, 8C). Caspase-8 is a suppressor of the RIP1 - RIP3 complex and necroptosis can be triggered when caspase-8 level was decreased [24, 29]. Thus, we also tested the protein level of cleaved caspase-8. Compared to sham group, cleaved caspase-8 was significantly decreased after 72 h of SAH (*P*<0.01 vs sham, Figure 8D), but Celastrol treatment prevented the degradation of it (*P*<0.01, Figure 8D).

### Celastrol reduced the amount of PI-positive cells after SAH

**Figure 9:**
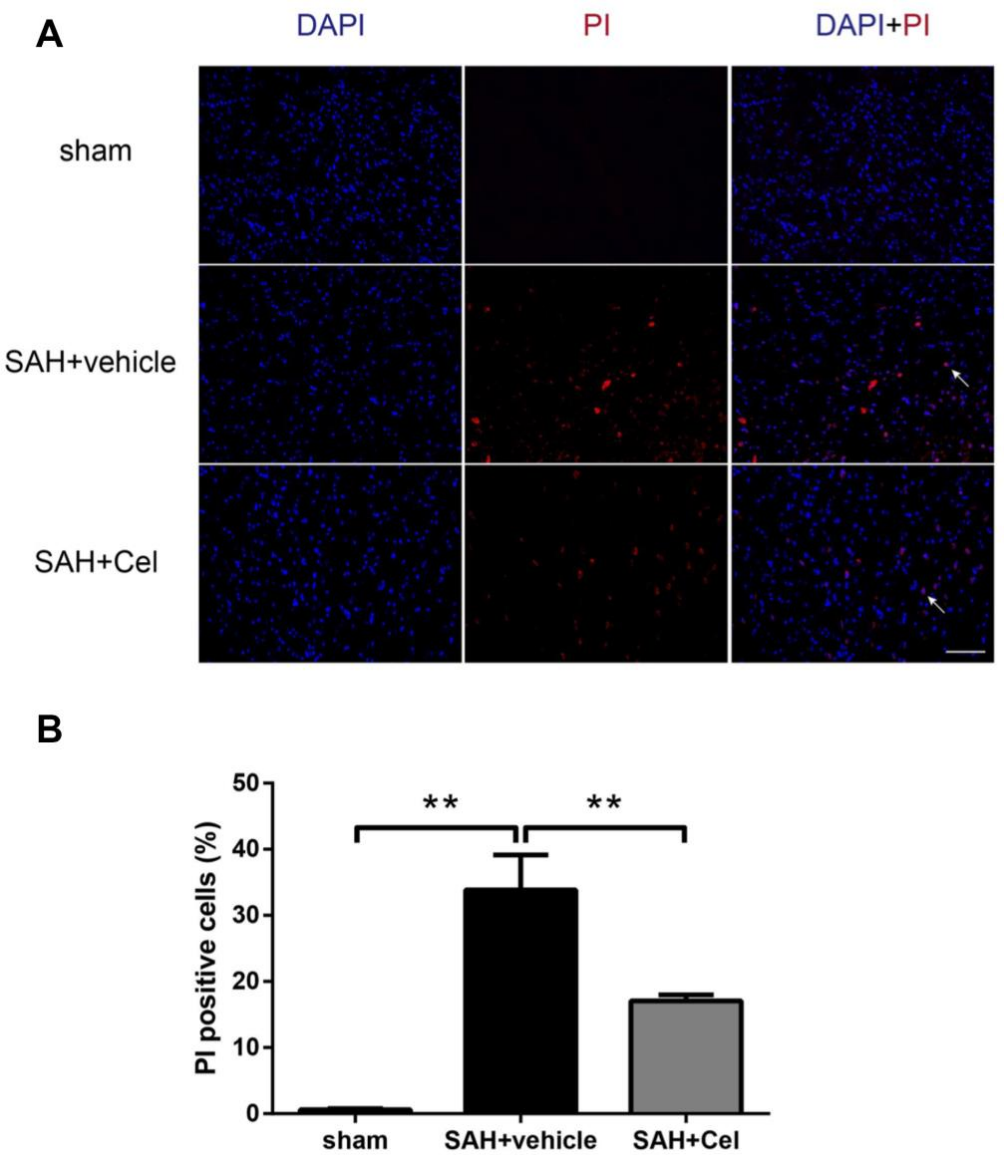
**Effects of celastrol on cell injury in the ipsilateral basal cortex at 72 h after SAH induction.** (**A**) Representative microphotographs showed the co-localization of DAPI (blue) with PI (red) positive cells in the ipsilateral basal cortex at 72 h after SAH induction. (**B**) Quantitative analysis of PI positive cells at 72 h after SAH induction. Data were presented as mean±SEM. n = 6. **P < 0.01. Scale bar = 100 μm.

In order to evaluate the effects of celastrol in preventing neural cell death, PI was used to identify the plasma membrane-ruptured cells. As shown in Figure 9A–9B, the amount of PI-positive cells was notably increased in the basal cortex of the ipsilateral hemisphere at 72 h after SAH (*P*<0.01 vs sham). Treatment with celastrol significantly reduced the percentage of PI-positive cells (*P*<0.01 vs SAH + vehicle).

## DISCUSSION

For the first time, our study found that celastrol, a botanical compound, could act as a neuroprotective agent on EBI after SAH by improving neurological function, alleviating brain swelling, reducing T2 lesion volume and ventricular volume. The possible mechanisms might be through preventing the distribution of tight junction proteins, alleviating BBB disruption, attenuating neuroinflammation and down-regulating the level of MMP-9. Moreover, celastrol suppressed the RIP3/MLKL-mediated cell necroptosis after SAH.

Nature compounds and their derivatives are good candidates for novel pharmacotherapies. Among them, triterpenoids comprise the largest group of botanical compounds [30]. Plant-derived triterpene celastrol has been proved to be a potent anti-tumor agent to a variety kind of malignant tumors, such as breast cancer [31], lung cancer [32], prostate cancer [7] and leukemia [33, 34]. Besides, celastrol was also known to be effective in many autoflammatory diseases, such as adjuvant-induced arthritis (AA) [35] and inflammatory bowel disease (IBD) etc [24]. The anti-tumor and anti-inflammatory functions of celastrol are based on its multi-target effects on diverse oncogenic or immune signaling pathways. For instance, celastrol acts as inhibitor of heat shock protein (HSP) 90 and proteasome [36], affecting NF-κB signaling pathway [37], and inhibiting the phosphorylation of ERK, JNK and STAT3 [35]. In recent years, neuroprotective effects of celastrol have been studied in several kinds of brain insults [13–15]. Nevertheless, to the best of our knowledge, there is no study reported about celastrol’s effects on SAH. Our study has discovered the neuroprotective attributes of celastrol after SAH, especially on attenuating brain swelling and protecting BBB from disruption. These results were consistent with previous study regarding celastrol’s effects of regulating tight junction integrity in murine brain endothelial bEnd3 cells [17].

As a fatal disease, there are very few effective pharmacotherapeutic strategies on treating SAH. Patients who suffer from SAH usually have a high mortality rate, or possible neurological deficits if survive [1, 2]. There are many animal models trying to simulate the pathological changes after SAH. Among them, the endovascular filament puncture model is the best to mimic the sudden increase of intracranial pressure when SAH happens due to a ruptured aneurysm [38, 39]. The high mortality rate (about 40%) in this study also reflects the severity of SAH caused by this method and also in line with reality. In the past, delayed cerebral vasospasm was considered as the major factor of unsatisfied prognostic outcome after SAH. Nonetheless, after the unsuccessful attempts of several clinical trials using anti-vasospastic drugs to treat SAH patients [40, 41], researchers turned their interests to EBI in the first 72 h after SAH [4]. The mechanisms of EBI include the sharp rise of intracranial pressure and the decrease of cerebral blood flow, which could cause BBB disruption, brain edema, neuroinflammation and cell death. Brain edema contributes to pathological changes including brain swelling and neuronal cell death [18], and has been recognized as an independent risk factor of poor prognosis after SAH [42]. Brain water content is widely used to access brain edema. But it is an invasive detection and only reflects the water content of the brain parenchyma. In this study, a 3.0-T MRI was used to evaluate the brain swelling levels, T2 lesion volumes and the ventricular volumes of rats in each group, because it was noninvasive and more accurate. Previous studies have illustrated that T2 lesion volume and ventricular volume were associated with neurological functions and the severity of SAH [43, 44], which is in line with the results of our study. The celastrol-treated SAH group showed decreased T2 lesion volume and ventricular volume as well as improved neurological scores than the vehicle group. These results consist with the decrease of brain water content and the improvement of neurological function after celastrol treatment in the present study and the previous studies about TBI and ischemic brain injury [15, 16]. Despite the obvious beneficial effects of celastrol on improving neurological function, decrease of the mortality rate was not observed. Most death of the animals happened in the first 1 h after SAH induction because of the drastic uprising of the intracranial pressure, and obviously, celastrol had limited effects during this acute change. But it is still possible to be used as an add-on therapy to improve the life quality and long-term prognosis of SAH patients.

Previous studies have found that celastrol also had the ability to down-regulate NF-κB-mediated MMP-9 level in breast cancer cells [45] and fibroblast-like synoviocytes in rheumatoid arthritis [46]. As BBB disruption caused by the degradation of tight junction proteins after stroke was mainly mediated by MMP-9 [26], and inflammatory cytokines were considered to be the main activators of MMP-9 [47]. Therefore, we also detected the level of MMP-9 and proinflammatory cytokines IL-1β, IL-6 and TNF-α 72h after SAH. The results indicated that celastrol could also down-regulate MMP-9 and inhibit neuroinflammation in EBI after SAH, so as to protect tight junction integrity.

Necroptosis is a newly discovered form of inflammatory cell death mediated by RIP3/MLKL signaling pathway [48]. During programmed cell death, RIP1 binds with RIP3 to form the necrosome [48, 49]. MLKL also plays a pivotal role in this process. After phosphorylated by RIP3, it forms an oligomer and directly disrupts membrane integrity [50]. To investigate celastrol’s effect on necroptosis after SAH, we examined the protein levels of RIP3 and MLKL in the ipsilateral cortex in each group. Celastrol treatment significantly down-regulated RIP3 and MLKL levels, compared to SAH + vehicle group. PI was used to identify the necrotic cell death [23, 51]. PI-positive cells was much less in SAH + Cel group, which supports celastrol’s effect on protecting neuronal cells from necrotic cell death. The protein level of cleaved caspase-8 was also measured, as caspase-8 can suppress the RIP1 - RIP3 complex and necroptosis can be triggered when caspase-8 is degraded [24, 29]. In this study, celastrol markedly up-regulated cleaved caspase-8 level after SAH, which was consistent with previous study in ulcerative colitis [24]. These data indicated that celastrol could act as a necroptosis suppressor in EBI after SAH.

## CONCLUSIONS

Our study reported, for the first time, that celastrol had neuroprotective effects on EBI after SAH by attenuating brain swelling and protecting BBB, which might due to the inhibition of MMP-9 and neuroinflammation. Additionally, celastrol also prevented necroptosis after SAH by inhibiting the RIP3/MLKL pathway. Our data suggest that celastrol deserves to be further investigated in clinical trials and could be developed as a therapeutic agent in SAH management.

## MATERIALS AND METHODS

### Animal study design

In total, 61 adult male Sprague-Dawley (SD) rats (300-320g, Slac Laboratory Animal Co., Ltd., Shanghai, China) were utilised in this study. All animal experiments and procedures were in accordance with the Guide for the Care and Use of Laboratory Animals of the National Institutes of Health. This study was approved by the Institutional Animal Care and Use Committee of Zhejiang University. Sixty-one SD rats were randomly divided into three groups: the sham group (n=14), SAH + vehicle group (n=24) and SAH + Cel group (n=23). The rat SAH model (endovascular perforation) was established as previously described [39]. The sham group underwent the same modeling procedure as the SAH group but without the intracranial arterial perforation. The SAH + vehicle and SAH+Cel group were treated with vehicle and celastrol, respectively. Based on previous studies [22, 52–54] and our own data(unpublished) regarding the time course of RIP3 and MLKL protein level after SAH, all the end points in this experiment were set as 72 h after SAH. In every group, 6 rats were used for MRI scanning and measurement of protein levels, 6 animals were used for PI labeling, and the other two rats were used for IHC.

### Drug administration

Celastrol purchased from Selleck Chemicals (Houston, TX, USA) was dissolved in DMSO and further diluted in sterile saline to a final concentration of 1%. 3 mg/kg celastrol (about 1 ml), which was then administrated through intraperitoneal injection immediately after SAH induction. The dose and time of celastrol treatment were based on a previous study [16]. Both sham group and SAH + vehicle group received the same amount of vehicle intraperitoneally as SAH+Cel group after SAH induction as study control.

### Neurological score and SAH grade evaluation

Neurological score evaluation were based on a previous system on 24, 48 and 72 h respectively after SAH [55]. The evaluation system consisted 6 different tests, and each test was scored as either 0-3 or 1-3, with the total score ranging from 3 to 18. The detailed neurological function evaluation system was shown in Supplementary Table 1. Randomized behavioral task sequence of the animals was used. When the rats were sacrificed, the severity of SAH was graded using an SAH grading system [56]. All the tests and SAH grades were evaluated by the same observer.

### MRI and measurements

At 72 h after SAH induction, MRI was performed with a 3.0-T GE Discovery MR750 scanner (General Electric Company, USA). Rats had T2 fast spin-echo sequences using a field of view of 60×60 mm, matrix of 256×256 and 9 coronal slices (2-mm-thick). All the MRI data was analyzed and evaluated by the same observer using ImageJ software. Brain swelling measurement was based on all 9 sections [57], and the value was calculated using the following formula: ((ipsilateral hemisphere volume - contralateral hemisphere volume)/ contralateral hemisphere volume) × 100% [58]. Ventricular volume was measured from the frontal horn of lateral ventricle to the foramen of Luschka as previously described [43], and was calculated using the following formula: Σ(A_n_ + A_n + 1_) × d / 2, where A is the ventricular area while d is the distance between sections. T2 lesion volume was identified as previously described [44]. In brief, a pixel was considered as abnormal if the value was larger than the mean plus twice the standard deviation of the mean value of contralateral hemisphere, and the T2 lesion volume was presented as the volume ratio to the ipsilateral hemisphere.

### Western blot analysis

WB was conducted as previously described [59]. The rats’ brains were resected and the left basal cortical samples containing blood clots were weighed and homogenized. After that, these samples were centrifuged for 10 minutes at 1000 g (4° C). The precipitate was discarded while supernatants were further centrifuged. Protein concentration was quantified by the detergent-compatible protein assay kit (Bio-Rad, Hercules, CA, USA). Equal amount of protein (40 μg) was mixed well with loading buffer, respectively, and denatured for 5 min at 95° C. After that, protein samples were loaded into SDS-PAGE gels for separation then transferred on polyvinylidene fluoride (PVDF) membranes. The membranes were subsequently blocked and probed with the primary antibodies overnight at 4° C. Antibodies used in this study and the dilutions were as follows: Albumin (Bethyl Laboratories A90-134A, 1:5000), ZO-1 (Santa Cruz SC-10804, 1:2000), Occludin (Santa Cruz SC-5562, 1:2000), Claudin-5 (Santa Cruz SC-28670, 1:800), MMP-9 (Abcam ab38898, 1:5000), IL-1β (Santa Cruz SC-23459, 1:800), IL-6 (Abcam ab9324, 1:2500), TNF-α (Abcam ab6671, 1:1000), RIP3 (Novus NBP1-77299, 1:1000), MLKL (Santa Cruz SC-165025, 1:500), caspase-8 (GeneTex GTX-110723, 1:3000), and β-actin (Abcam ab8226, 1:5000). The membranes were incubated with secondary antibodies (1:5000) for 1 h at room temperature (RT). The protein bands were visualised using X-ray film, quantified by ImageJ software (NIH) and normalized to β-actin.

### Immunohistochemistry

IHC was performed as previously described [60]. The rats’ brains were removed then dehydrated. Coronal frozen sections (7 μm thick) were obtained as described previously. The sections were incubated with goat anti-mouse albumin antibody (Bathyl Laboratories A90-134A, 1:500) for 2 h at RT and washed with PBS, then incubated with secondary antibody for 1h at RT. Immuno detection was performed using a DAB peroxidase substrate kit SK-4100 (Vector Laboratories, Inc., Burlingame, CA, USA). Images were acquired by Olympus BX41 microscope (Olympus, Tokyo, Japan).

### PI labeling

At 71 h after SAH induction, PI (10 mg/mL; Sigma-Aldrich, St Louis, MO, USA) was diluted in sterile normal saline and injected intraperitoneally (30mg/kg). The rats were sacrificed 1 h thereafter and brains were removed and soaked in 4% formaldehyde for 48 h at 4° C, then dehydrated in 30% sucrose solution until the brain sank to bottom (approximately 2 days). Coronal frozen sections (7 μm thick) were cut with the use of tissue-freezing media at 150-200 μm intervals near the optic chiasma. PI-positive cells were quantitated in the left basal cortex from 200× cortical fields in three brain sections per rats under a fluorescence microscope (Olympus, Tokyo, Japan), which were then evaluated by the same observer.

### Statistical analysis

Statistical analyses were performed using SPSS (version 22.0) and Prism (version 6.0) software. Data were expressed as mean ± SEM. Comparison among the groups were analyzed using one-way analysis of variance (ANOVA) followed by Tukey’s multiple comparison test. P value less than 0.05 was considered as statistically significance.

## Supplementary Material

Supplementary Table 1
