# Supplementary material for: Celastrol protects against early brain injury after subarachnoid hemorrhage in rats through alleviating blood-brain barrier disruption and blocking necroptosis
Source: Aging (Albany NY). 2021 Jun 28;13(12):16816–33. doi: 10.18632/aging.203221 (PMC8266331; doi:10.18632/aging.203221)
Supplement: Supplementary Table 1 [file aging-13-203221-s001.pdf]

SUPPLEMENTARY TABLE

Supplementary Table 1. Evaluation of the neurological score after SAH.

Test	Score			
	0	1	2	3
**Spontaneous activity (in cage for 5 min)**	No movement	Barely moves	Moves but does not approach at least three sides of cage	Moves and approaches at least three sides of cage
**Symmetry in limb movement**	Right side: no movement	Right side: slight movement	Right side: moves slowly	Both sides: move symmetrically
**Forepaw outstretching**	Right side: no outreaching	Right side: slight movement to outreach	Right side: moves and outreaches less than left side	Symmetrical outreach
**Climbing**	\	Fails to climb	Right side is weak	Normal climbing
**Response to touch on either side of trunk**	\	No response on right side	Weak response on right side	Symmetrical response
**Response to vibrissae touch**	\	No response on right side	Weak response on right side	Symmetrical response
